# Development of a screening model for APL using cell population data and deep learning-extracted WBC scattergram features

**DOI:** 10.1186/s12885-025-15034-7

**Published:** 2025-11-07

**Authors:** Qi Cai, Bo Ye, Wenbo Zheng, Shihong Zhang, Jingxian Zhang, Yimin Shen, Donglan Yao, Huihui Zhang, Zhixi Huang, Jian Hu, Yushuai Ma, Jianbiao Wang, Yong Wang

**Affiliations:** 1 Department of Clinical Laboratory, School of Medicine, Ruijin Hospital, Shanghai Jiaotong University, Shanghai, 200025 China; 2 Clinical Department (IVD), Shenzhen Mindray Bio-Medical Electronics Co., Ltd, Shenzhen, 518057 China; 3 Department of Clinical Laboratory, The First Affiliated Hospital Sun Yat-sen University, Guangzhou, 510062 China; 4 Department of Clinical Laboratory, The First Affiliated Hospital of Soochow University, Suzhou, 215006 China; 5 Department of Clinical Laboratory, The First Affiliated Hospital of Zhengzhou University, Zhengzhou, 450008 China

**Keywords:** Blood cell scattergrams parameters, Acute promyelocytic leukaemia, Machine learning

## Abstract

**Background:**

Acute promyelocytic leukemia (APL), a high-risk subtype of acute myeloid leukemia, necessitates rapid diagnosis upon hospital admission to mitigate early mortality. Current diagnosing approaches relying on time-consuming genetic testing or morphological expertise are particularly challenging in resource-limited settings. Herein, this study introduces a novel machine learning approach leveraging routine lab data to enable immediate APL suspicion, offering a new diagnostic possibility for under-resourced hospitals.

**Methods:**

We developed a two-stage machine learning model using multi-center retrospective data. The cohort included 94 confirmed APL patients (2020–2024) from three tertiary hospitals, with an external validation set (*n* = 541) from an independent center. Using four VGG-16 networks, we extracted APL-specific 3D scatterplot features from DIFF and WNB channels of routine blood tests. These features were then fed into an optimized random forest classifier-scatterplot (RFC-S) model, refined via recursive feature elimination and threshold tuning.

**Results:**

The RFC-S model achieved near-perfect discrimination, with an AUC of 0.9893 in the test set and 0.9979 in external validation. It maintained 98.15% sensitivity and 95.52% specificity—outperforming conventional methods. SHAP analysis confirmed that key scattergram-derived features (e.g., *N_APL_Ratio_YZ*) drove predictions. Critically, the model requires no additional tests, making it deployable even in low-resource clinics.

**Conclusions:**

The RFC-S model represents an innovative approach to APL screening by combining deep learning-derived scattergram features with routine blood parameters. This two-stage methodology achieves high diagnostic accuracy (AUC > 0.98) while maintaining computational efficiency. Importantly, the model’s ability to utilize existing laboratory data without requiring additional tests makes it particularly valuable for resource-constrained settings where access to genetic testing or hematological expertise may be limited. Our findings suggest this approach could serve as a practical tool for early APL identification, potentially reducing diagnostic delays in diverse clinical environments.

**Supplementary Information:**

The online version contains supplementary material available at 10.1186/s12885-025-15034-7.

## Introduction

Acute promyelocytic leukemia (APL), characterized by the PML-RARA fusion oncogene [1], represents 5–10% of acute myeloid leukemia (AML) cases with distinct clinical urgency due to rapid progression and life-threatening coagulopathy [2, 3]. Although combination therapy with all-trans retinoic acid (ATRA) and arsenic trioxide induces PML-RARA degradation, achieving 80% cure rates in APL [4–6]. Despite therapeutic advancements in APL management, the early death (ED) rate remains alarmingly high at 32% [7, 8]. Critically, 35% of ED cases involve patients never administered ATRA, primarily due to mortality from undiagnosed disease prior to treatment initiation [7]. These findings underscore the pivotal role of early diagnostic strategies in mitigating ED risk.

The diagnosis of acute leukemia necessitates a comprehensive analytical workflow integrating cytomorphology, immunophenotyping, cytogenetic analysis, and molecular profiling, typically requiring ≥ 3 days for completion. Artificial intelligence (AI) demonstrates potential in medical applications, such as identifying novel antimicrobial compounds to address resistance and improving diagnostic accuracy for hematologic malignancies through machine learning models [9–11]. The identification of abnormal promyelocytes with characteristic bundled Auer bodies in peripheral blood smears represents a pathognomonic morphological hallmark for presumptive APL diagnosis. However, this rapid presumptive diagnosis relies on morphological expertise that is often unavailable in resource-constrained settings – a disparity strongly correlated with elevated ED rates in low-income regions [12, 13]. Furthermore, routine morphological screening is not universally applied to all clinical specimens. To address this gap, haematology analyzer-based strategies have been explored. Prior studies developed artificial neural network (ANN) models using complete blood count (CBC) parameters and cell population data (CPD) for APL screening [14, 15]. Subsequent work employed convolutional neural networks (CNNs) to detect high-dimensional morphological signatures in white blood cell differential scattergrams, demonstrating improved sensitivity over ANN approaches [16]. Nevertheless, CNN-based frameworks exhibit two key limitations: (1) intrinsic black-box nature with limited interpretability; (2) their high computational demands require specialized GPU hardware, making them unsuitable for haematology analyzers due to tight resource constraints (compute, power, thermal, and space).

In this study, given the heterogeneous scattergram patterns observed in APL patients’ hematological analyses, we developed a dual-phase analytical framework: (1) VGG-16 convolutional neural networks were employed to detect morphometric anomalies in DIFF/WNB channel scattergrams, establishing twenty novel quantitative parameters to characterize APL-specific regions; (2) these CNN-derived parameters were integrated with conventional hematological indices (routine blood parameters/CPD) to construct an interpretable screening model for frontline APL screening.

## Materials and methods

### Data collection

From July 2020 to April 2024, data from 2,682 participants were retrospectively collected from three hospitals: the First Affiliated Hospital of Sun Yat-sen University (July 2020 to July 2023), the First Affiliated Hospital of Soochow University (January 2021 to April 2022), and Ruijin Hospital affiliated with Shanghai Jiaotong University School of Medicine (February 2023 to April 2024). This cohort included 94 APL patients, 765 AML patients excluding APL, 323 acute lymphocytic leukaemia (ALL) patients, and 750 healthy controls (HCs). For model development, data from the First Affiliated Hospital of Soochow University and the Ruijin Hospital were used. These data were divided into training and test sets in an 8:2 ratio. In addition, we conducted external validation using data from the First Affiliated Hospital of Sun Yat-sen University (July 2020 to July 2023), including those from 20 APL patients, 220 AML patients, 101 ALL patients, and 200 HCs (Supplementary Fig. 1). This study was approved by the institutional review board of Shanghai Jiaotong University School of Medicine Ruijin Hospital (ethics approval number 2021 (72)). Informed consent was waived because the research utilized only de-identified residual samples obtained during routine clinical testing, and the study posed no additional risk to patients.

Among the acute leukaemia patients, only adult patients diagnosed and classified according to the World Health Organization (WHO)-recommended morphology, immunology, molecular and cytogenetics diagnostic criteria were included in this study [17]. All APL cases were confirmed to have PML-RARA fusion. The healthy controls in this study were hospital examination patients with all complete blood count parameters within normal ranges. The information of all participants were recorded. The cell analysis data of all patients were output by a Mindray BC-6800 Plus haematology analyser. We obtained four fluorescence-related scattergrams: the side scatter (SS)- fluorescence (FL) and forward scatter (FS)-FL scattergrams of the DIFF channel and those of the WNB channel, with scattergram dimensions of 256 × 256 × 3.

### Extracted APL-specific 3D scatterplots features

#### Identify APL-specific 3D scatterplots regions

The VGG16 architecture employs a modular design comprising stacked 3 × 3 convolutional layers and 2 × 2 max-pooling operations, enabling hierarchical feature extraction while maintaining computational efficiency. This configuration progressively increases network depth and receptive field coverage, allowing effective detection of APL-associated cellular patterns (e.g., abnormal granularity, cluster distributions) within dual-channel 3D-DIFF and 3D-WNB scattergrams.

**Fig. 1:**
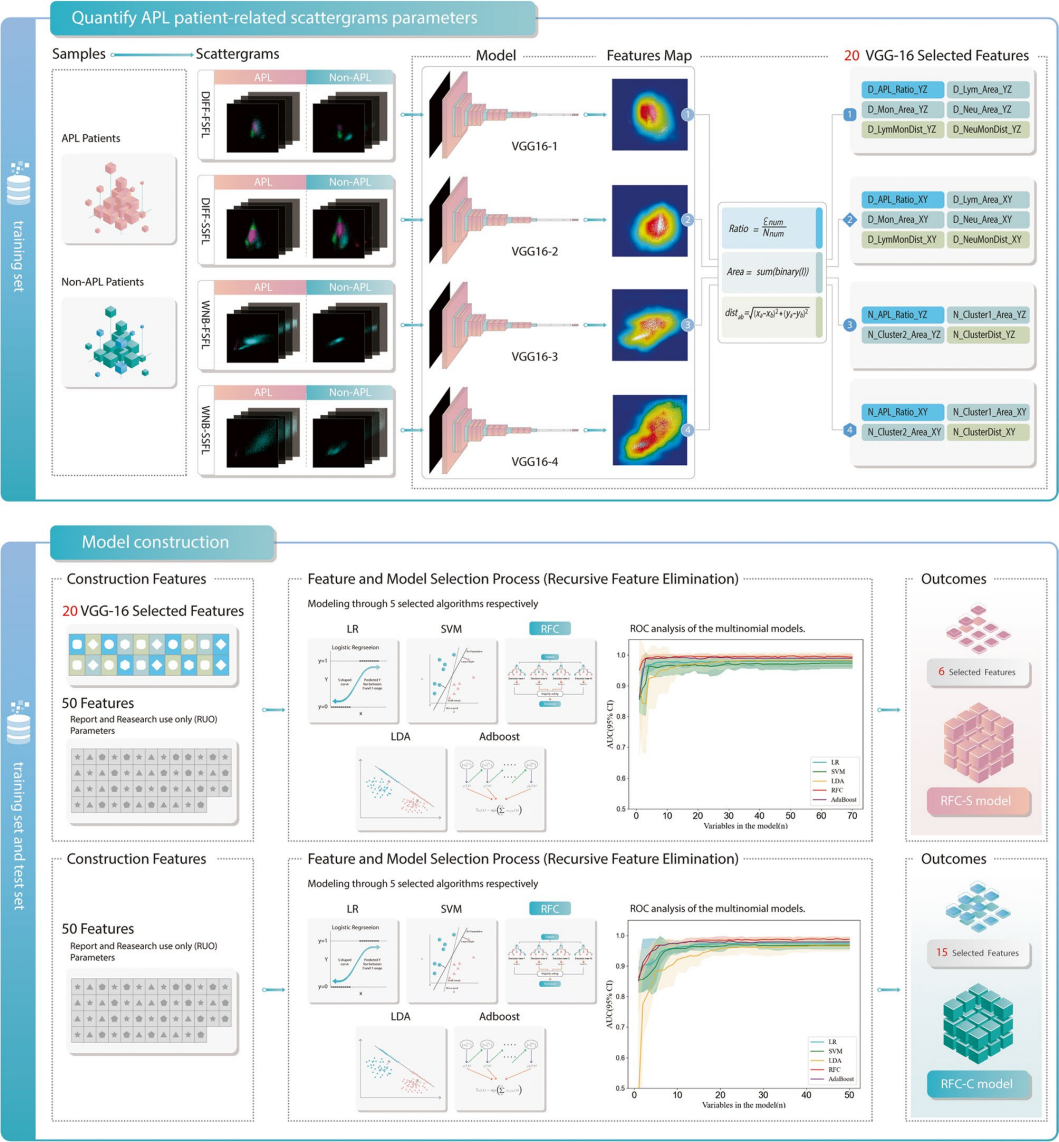
Flow chart for modeling. Deep Feature Extraction: Four VGG-16 convolutional neural networks (CNNs) analyze 3D scattergrams from DIFF and WNB channels (SS-FL and FS-FL perspectives) to identify APL-specific morphological patterns. Gradient-weighted class activation mapping (Grad-CAM) localizes regions of interest (ROIs), which are quantified into 20 novel scattergram parameters (e.g., particle count, area, spacing). Interpretable Model Construction: The CNN-derived parameters are integrated with 50 routine blood cell parameters (CBC/CPD) to train five machine learning classifiers. Recursive feature elimination (RFE) selects optimal features (e.g., *N-APL-Ratio-YZ*, PCT) for two distinct models: RFC-S (Random Forest Classifier-Scattergram): Combines both CNN-extracted scattergram features and routine parameters; RFC-C (Random Forest Classifier-CPD): Utilizes only conventional cell population data (50 parameters) as a comparative baseline. Abbreviations: APL (acute promyelocytic leukemia), SS-FL (side scatter-fluorescence), FS-FL (forward scatter-fluorescence), CPD (cell population data), CNN (convolutional neural network)

Therefore, we used the classic VGG16 CNN architecture to differentiate the four types of scattergrams (SS-FL and FS-FL of the DIFF channel, and SS-FL and FS-FL of the WNB channel) of APL samples from those of other samples. We input these four scattergrams into four VGG16 networks for APL identification training (Fig. 1). To account for differences between haematology analyzer instruments and reagent lots and to improve the generality of the model, we preprocessed the data through image normalization and data enhancement. The normalization of scattergrams involves resizing all images to a uniform dimension using Open CV’s resize function, while the intensity is normalized using min-max normalization to linearly scale pixel values to the [0, 255] range. For scattergram enhancement, a logarithmic transformation is applied to the SSC (side scatter) signals to improve the differentiation between particle clusters. The network was subsequently trained in an end-to-end manner using a backpropagation algorithm and a stochastic gradient descent optimizer. The training hyperparameters were set as follows: learning rate, 0.000001; momentum, 0.9; weight decay, 0.0001; number of iterations, 100; and batch size, 10. During training, we used the cross-entropy loss function to measure the difference between predicted values and actual labels and continuously adjusted the network weights to minimize the loss. Finally, the APL classification accuracy of the four VGG16 networks on the training set all exceeded 82%.

#### Locate APL-specific 3D scatterplots regions

To gain deeper insights into how the four VGG16 models identify APL, gradient-weighted class activation mapping (Grad-CAM) technology was employed. The heatmap’s colour intensity represents the model’s attention to specific regions in the image, with warmer (red) colours indicating regions of greater importance for APL identification.

#### Quantify APL-specific 3D scatterplots regions

As illustrated in Fig. 1, we performed a quantitative analysis of the four scattergrams (DIFF and WNB channels) for APL samples by analysing the regions of interest (ROIs) in Grad-CAM heatmaps. Specifically, we utilized three image-processing metrics to characterize these scatter plots: ROIs particle count, ROI particle population area, and particle population spacing. These metrics were chosen because they directly reflect morphological changes between cells or cell clusters and are relatively less prone to interference compared to other features, such as texture. The detailed calculations are provided in the Supplementary Material.

### Construction of the machine learning models

We quantified APL-specific 3D scatterplots regions identified by the VGG16 model through 20 scattergram parameters, and input 50 blood cell parameters, as shown in Supplementary Table 1 (70 blood cell parameters), into five machine learning models. These classifiers include logistic regression (LR), support vector machine (SVM), linear discriminant analysis (LDA), random forest classifier (RFC), and adaptive boosting (AdaBoost). For each model, the optimal parameters were found based on recursive feature elimination (RFE) and five-fold cross-validation, followed by a hyperparameter grid search for parameter tuning. For the RFC-S model (Constructing a Random Forest Classifier model using scattergram parameters), we set the number of decision trees to 300, class_weight to ‘balanced_subsample’, and max_features to the default value ‘auto’. In addition, we discarded 20 scattergram parameters related to APL and used the remaining 50 blood cell parameters to construct a RFC-C model (Constructing a Random Forest Classifier model using cell population data), we set the number of decision trees to 150, class_weight to ‘balanced_subsample’, and max_features to the default value ‘auto’. The performance of the two models was evaluated on the internal test set and the external validation set using the AUC, confusion matrix, specificity, and sensitivity, and the classification performance of the two models was further explained through SHapley Additive exPlanations (SHAP) analysis.

We performed data cleaning and normalization on the 70 blood cell parameters. Data cleaning was applied to remove duplicate values and input errors from the test samples to ensure data validity. Feature normalization was used to eliminate the difference in sample feature distribution caused by the inconsistencies in the gain and calibration coefficients of different instruments, avoid interference from outliers during model training. For blood cell feature normalization, all parameters were standardized using sklearn’s StandardScaler (Z-score normalization). The training set underwent fit_transform() to simultaneously calculate and apply the scaling parameters, while the test set only utilized transform() with the precomputed parameters to prevent data leakage. In this study, continuous variables were described using the mean (± standard deviation), and statistical analysis was conducted using Python 3.11 scikit-learn.

## Results

### Correlation analysis of APL-specific 3D scatterplots features

**Fig. 2:**
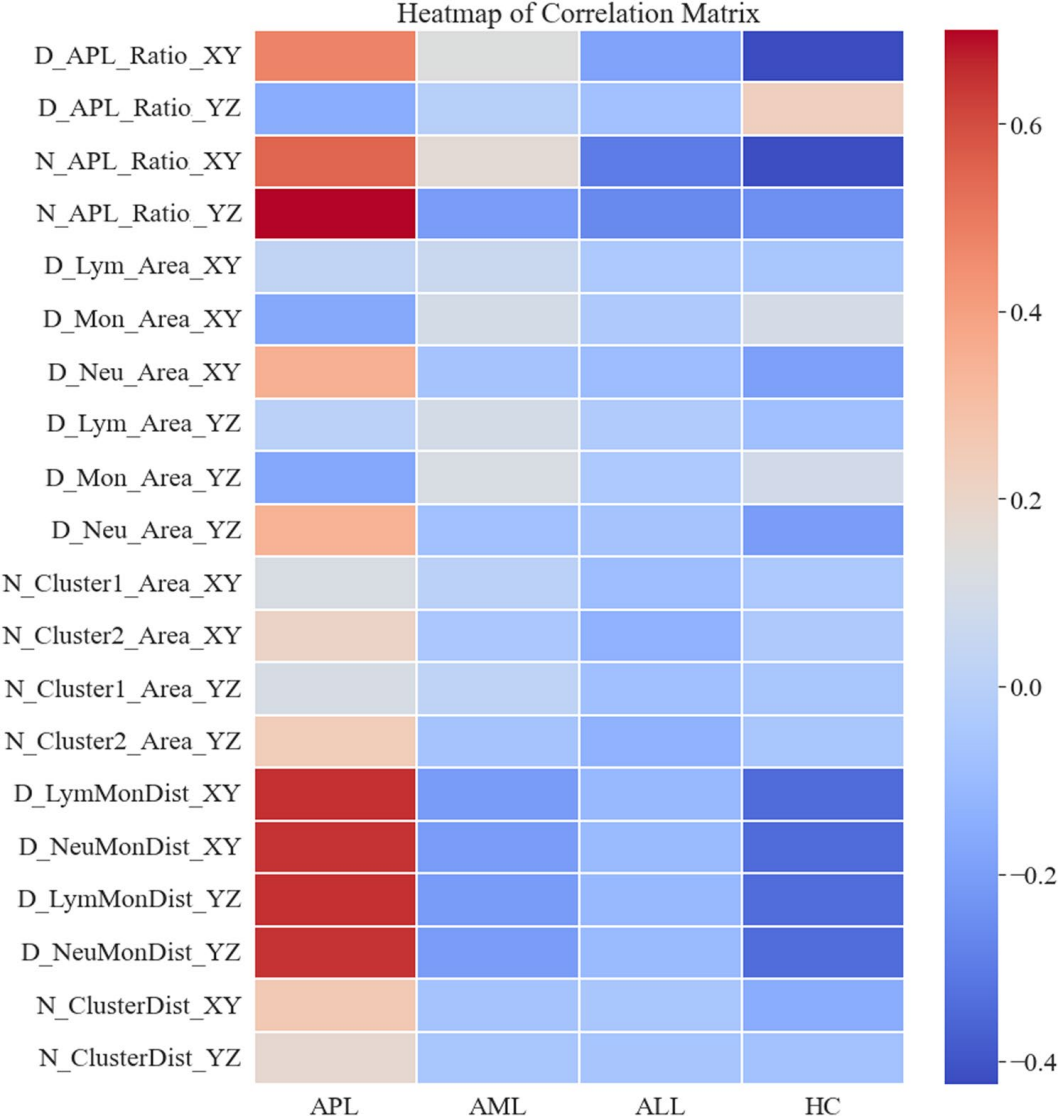
Differences in scatterplot parameters in different groups of patients. The heatmap of correlation matrix: color grading and clustering trends of 20 scattergram parameters among study groups. For heat map color grading ‘diverging Red to Blue’ scheme (for higher to lower values, respectively) was used. AML, acute myeloid leukemia; APL, acute promyelocytic leukemia; ALL, acute lymphoblastic leukemia; HC, healthy controls.

We constructed four VGG16 models to quantify the APL-specific regions in the 3D-DIFF and 3D-WNB scattergrams, as detailed in Method 2. Through heatmap analysis, we further explored the correlations between the 20 scattergram parameters we identified and APL. Parameters such as *N-APL-Ratio-YZ*, *D-LymMonDist-XY*, *D-NeuMonDist-XY*,* D-LymMonDist-YZ*, and *D-NeuMonDist-YZ* were all highly positively correlated with APL, while *D-APL-Ratio-YZ* was positively correlated in the HC group and negatively correlated in the APL group, as shown in Fig. 2. The correlation of all parameters across different disease groups can be seen in Supplementary Fig. 2.

### Model construction

**Fig. 3:**
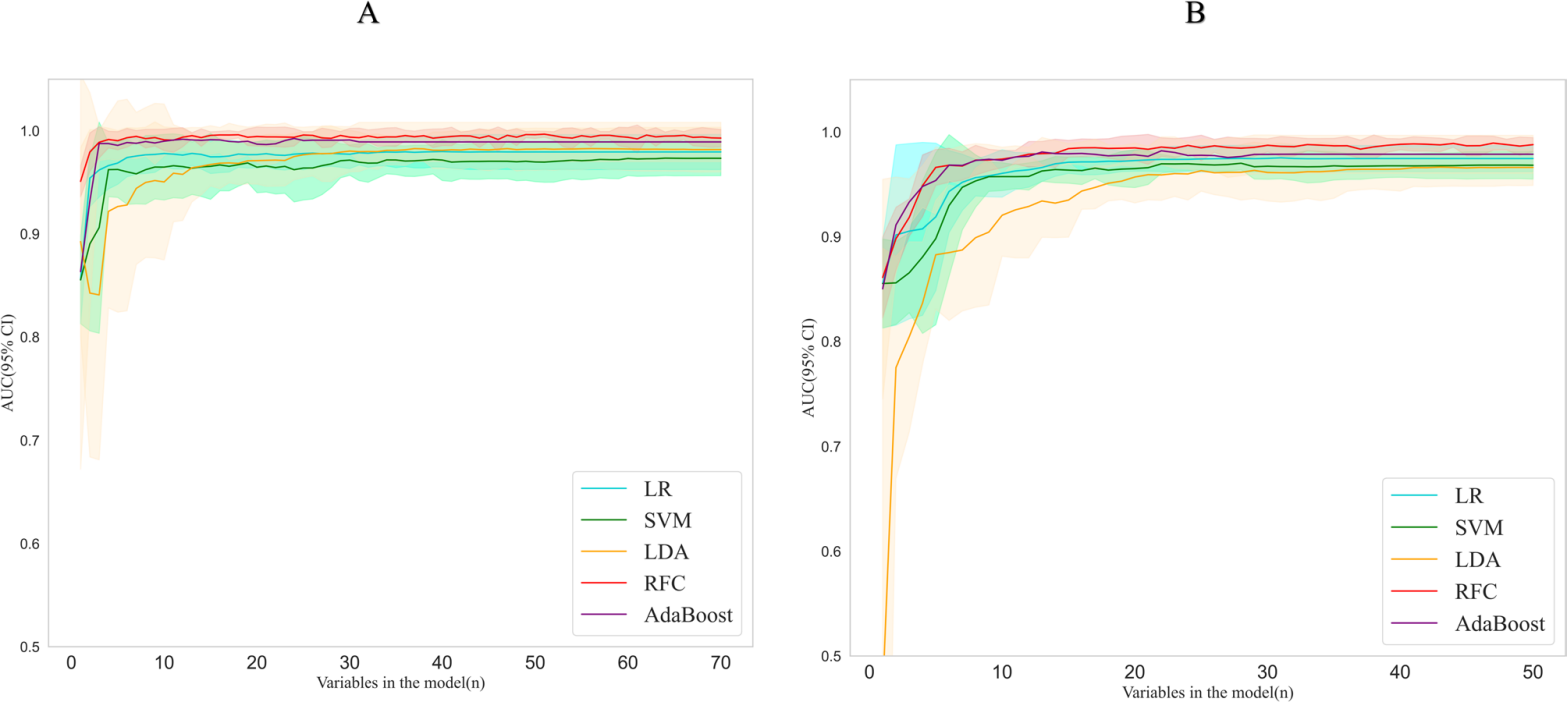
ROC analysis of the multinomial models. A shows the selection of variables for each model starting from the maximum number of variables (*n* = 70) and retaining the highest n-1 variables based on their importance in the model. B shows the selection of variables for each model starting from the maximum number of variables (*n* = 50) and retaining the highest n-1 variables based on their importance in the model. ROC: Receiver Operating Characteristic, AUC: area under the receiver operating characteristic curve, LR: logistic regression, SVM: support vector machine, LDA: linear discriminant analysis, RFC: random forest classifier, AdaBoost: adaptive boosting.

As shown in Fig. 3, the RFC model showed marginally better performance than the other models. When constructing the novel RFC-S model, recursive feature elimination based on cross-validation revealed that when the number of features was greater than 6, the receiver operating characteristic curve (ROC) performance of the four models did not significantly improve, and the ROC score decay rates were all less than 0.3%. However, when the number of features was less than 6, as the number of features decreased, meaning that the removal of each feature noticeably decreased the model prediction performance in Fig. 3A. For the RFC-C model, we found that when the number of features exceeded 15, the ROC performance of the five models did not improve significantly in Fig. 3B, with the ROC score decay rates similarly remaining below the 0.3% threshold.

Supplementary Fig. 3 shows that PLT and PCT ranked high in importance in the two models. The novel APL model was constructed using the top 6 feature parameters ranked in terms of feature importance (Supplementary Fig. 3 A), with four of them being APL-related scattergram parameters identified through the VGG16 network: *N-APL-Ratio-YZ*,* N-ClusterDist-YZ*,* D-APL-Ratio-XY*, and *D-Neu-Area-XY.* The RFC-C model was constructed using the top 15 feature parameters ranked in terms of feature importance in Supplementary Fig. 3B, among which the CPD parameter involved was *D-Neu-SFL-W*.

### Performance demonstration

**Fig. 4:**
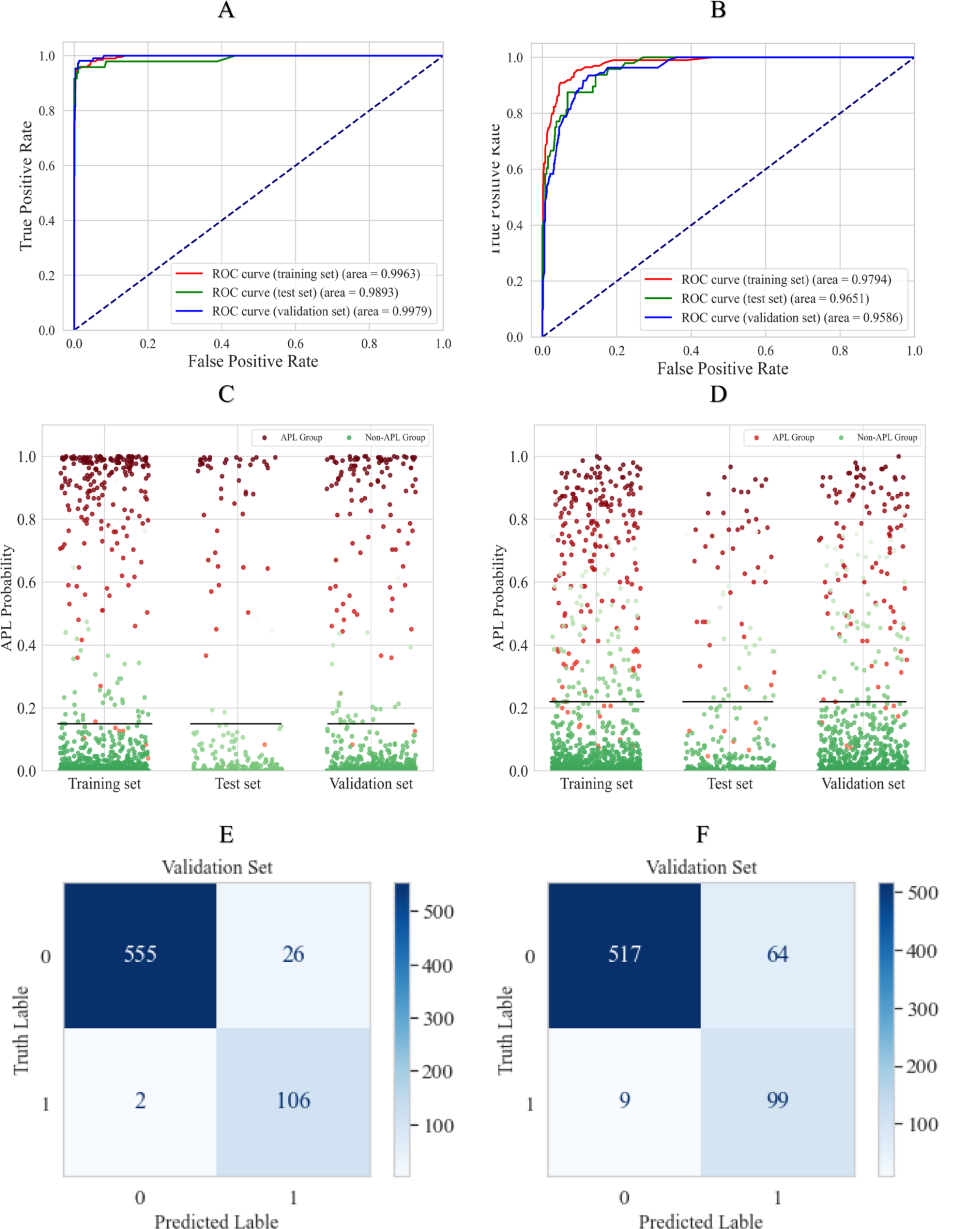
Performance demonstration of RFC-S model and RFC-C model. A shows the receiver operating characteristic curve (ROC) for the RFC-S and labels the area under the curve (AUC). B shows the ROC for the RFC-C and labels the AUC. C shows the probabilities of the RFC-S model predicting that it is an APL for different types of samples. D shows the probabilities of the RFC-C model predicting that it is an APL for different types of samples. E shows the confusion matrix of the RFC-S in external validation set. F shows the confusion matrix of the RFC-C in external validation set. APL, acute promyelocytic leukemia.

The RFC-S model and the RFC-C model perform well on both the training and validation sets, and have shown comparable performance on the external validation set. As shown in Fig. 4A, the area under the receiver operating characteristic curve (AUC) of the RFC-S model was 0.9963 and 0.9893 on the training and test sets, respectively, and reached 0.9979 on the external validation set. The RFC-S model screened for APL with 95.94% accuracy, 98.15% sensitivity, and 95.52% specificity in the external validation set (Table 1). Although the RFC-C model also performed well, its AUC on the external validation set was 0.9586, which was slightly inferior to that of the RFC-S model (Fig. 4B). The prediction performance of the two models on the different categories of samples in the three datasets is shown in Fig. 4C and D. The scatter points above the histogram boundary represent the higher probability that the model predicted them as APL. The RFC-S model was notably better at distinguishing between APL samples and other samples compared to the RFC-C model, which failed to distinguish many samples using the prediction threshold.

**Table 1:** RFC-S model and RFC-C model performance in three set

		Accuracy	Sensitivity	Specificity	PPV	NPV
**RFC-S** **model**	**Training set**	96.09%	96.46%	96.02%	81.97%	99.31%
	**Test set**	97.77%	95.83%	98.12%	90.20%	99.24%
	**Validation set**	95.94%	98.15%	95.52%	80.30%	99.64%
**RFC-C** **model**	**Training set**	92.17%	92.93%	92.03%	68.66%	98.58%
	**Test set**	90.13%	87.50%	90.60%	62.69%	97.57%
	**Validation set**	89.40%	91.67%	88.98%	60.74%	98.29%

The goal in constructing these models was to effectively identify APL patients from other samples through blood cell analysis. To this end, the sensitivity of the RFC-S model we constructed reached more than 95%. Only two samples were not identified by the RFC-S model in the external validation set, whereas the RFC-C model missed nine APL samples (Fig. 4E and F).

### Feature analysis of the novel APL model

**Fig. 5:**
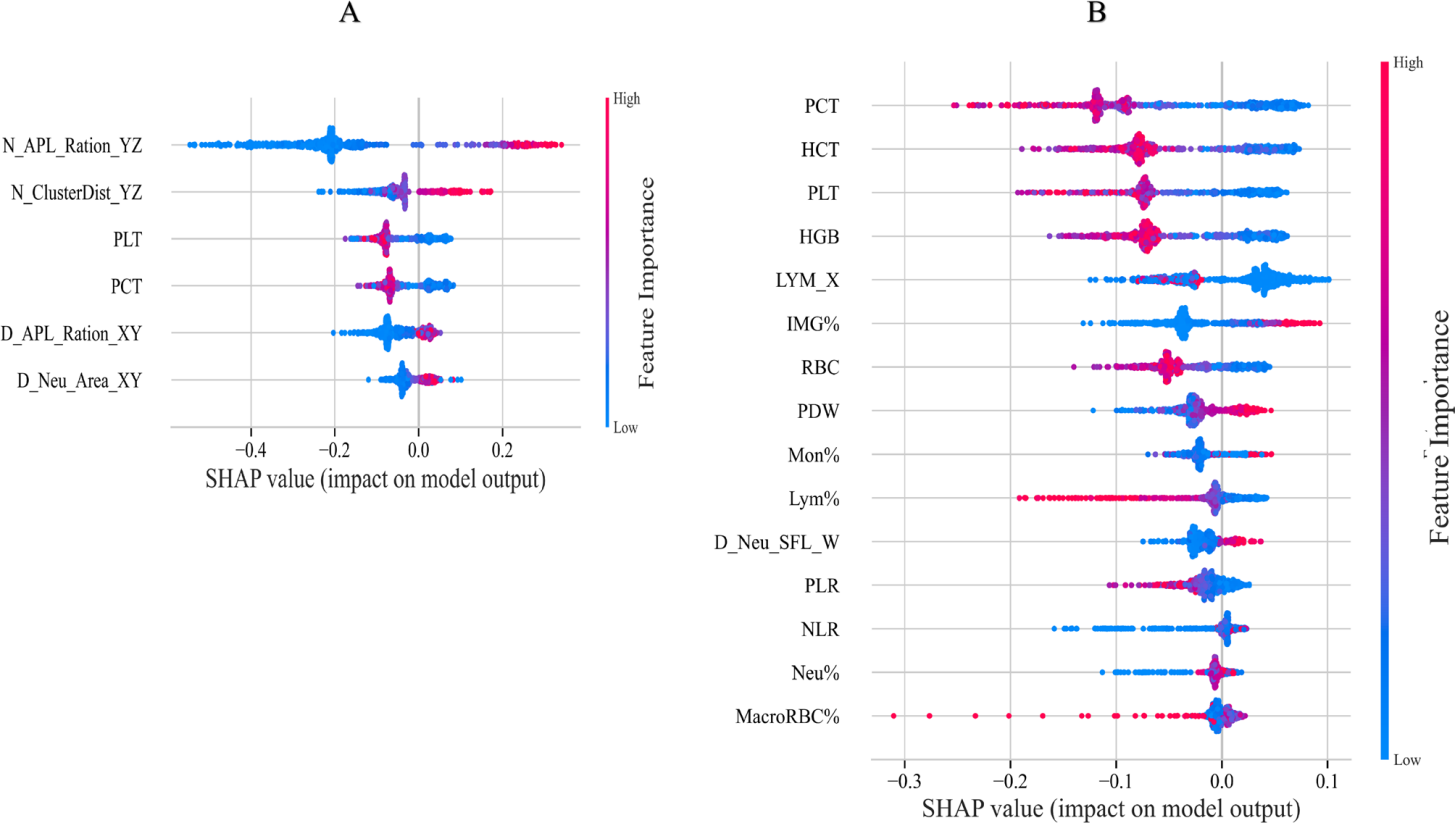
Sharpley additive interpretation (SHAP) analysis for RFC-S and RFC-C. SHAP analysis (A) for the 6 feature clusters derived by aggregating the correlation values (e.g., mean, minimum, and maximum) of specific features in the RFC-S model. SHAP analysis (B) for the 15 feature clusters derived by aggregating the correlation values (e.g., mean, minimum, and maximum) of specific features in the RFC-C model. Each dot corresponds to the SHAP value of the feature cluster for the APL risk score of a given case patient or control subject at a certain point in time. A feature’s SHAP value (x-axis) represents the contribution of the specific feature to the risk score, with positive values indicating a contribution that increases the risk score and negative values indicating a contribution that lowers the score. The location of the dot on the x-axis represents its SHAP value, whereas its color represents the cluster’s value (the actual value of the feature that is represented in the cluster), with red representing higher values (for features measured along a continuum) or affirmative responses (for binary features). The dots are piled up vertically to show their density. The feature clusters are sorted by their mean absolute SHAP values.

Next, in the RFC-S model (Fig. 5A), scattergram-derived features demonstrated superior discriminative power, with *N_APL_Ratio_YZ* (the proportion of APL-related particles in WNB channel’s FS-FL perspective) emerging as the most influential predictor. The clear separation of high and low risk clusters along this feature’s value range (SHAP values spanning − 0.4 to 0.2) confirms its strong diagnostic value. Notably, three of the top six features were APL-specific scatterplot parameters (*N_APL_Ratio_YZ*,* D_APL_Ratio_XY*,* D_Neu_Area_XY*), which collectively provided unique morphological information unavailable in conventional blood parameters.

By contrast, the RFC-C model (Fig. 5B) relied primarily on routine hematological indices, with platelet-related parameters (PCT, PLT) showing the highest importance. While these features achieved moderate discrimination (SHAP range: −0.3 to 0.1), their predictive power was substantially weaker than scattergram features in RFC-S. This explains the RFC-C model’s inferior performance, as conventional blood markers cannot capture the distinctive cellular morphology patterns that scattergram analysis detects.

## Discussion

Early detection of APL is extremely important due to the severe nature of the disease, making it critical to develop methods for early-stage identification. Previous studies have established machine learning models based on parameters such as fibrinogen, lactate dehydrogenase, platelet count, and patient age to predict the probability of APL. These models have achieved good results on external validation sets from different regions [13, 18]. However, a common issue with these models is missing data, as not every APL patient undergoes coagulation, biochemical, and blood cell analysis without a definite diagnosis. To make the model more widely applicable, many current studies have established models for the identification of APL samples solely based on blood cell analysis parameters [14, 15, 18], using AI to reveal the patterns of white blood cell changes in APL patients, thereby enabling stable classification [2]. Second, existing models rely on parameters that, while effective, are not unique to APL. Key parameters that play important roles in these studies, such as the mean corpuscular volume (MCV), platelet count, and mean corpuscular haemoglobin concentration (MCHC), also play important roles in identifying myelodysplastic syndromes and other haematological diseases because changes in blood flow characteristics in patients with haematological diseases affect the platelet count, red blood cell size, and haemoglobin heterogeneity [19–22]. These findings indicate that changes in these parameters are not specific to APL patients. Third, some studies have established CNNs to directly classify WDF scattergrams of APL patients from those of other patients. However, CNN-based models often require advanced hardware and offer limited interpretability. Therefore, these models cannot solve the problem of APL screening in regions lacking medical resources [16].

The French–American–British Cooperative Group (FAB) classifies abnormal promyelocytes into coarse granular, fine granular, and microgranular based on their size, and the morphological manifestations in the peripheral blood of APL patients are diverse. Previous studies relied on the WDF channel to detect changes in peripheral blood cells of APL patients. However, there are many types of scattergrams reflecting changes in white blood cells. Therefore, the present study used the VGG16 network to comprehensively capture white blood cell changes in APL patients across four scattergrams from dual channels and perspectives. Compared to the RFC-C model, we observed that the RFC-S model exhibited better performance on both internal and external validation sets. This disparity lies in the fact that the RFC-S model was constructed by utilizing scattergram features identified with the aid of a CNN. These scattergram features display a significantly high correlation with acute promyelocytic leukemia (APL). For example, *N-APL-Ratio-YZ* demonstrates a large differentiation between positive and negative samples, which can be clearly seen from the SHAP.

On the external test set, the RFC-S model missed only two APL samples, these samples had peripheral blood morphology results, and we found that the peripheral blood of this sample was dominated by neutrophil myelocytes and neutrophil metamyelocytes. According to the sample analysis of the false positives in the RFC-S model, we found that all five samples had acute leukaemia, including two samples with myelosuppression after chemotherapy and three samples with partially mature AML (M2 subtype). Promyelocytes with granules were also found in the peripheral blood of M2 samples and are difficult to differentiate from APLs, especially those microgranules, on the basis of peripheral blood morphology alone. The validation results revealed that the novel APL model effectively captured abnormal cell morphology in the peripheral blood of APL patients using blood cell scattergrams. We found that the RFC-C model missed six samples in the external validation set (four more than the RFC-S model did). Further analysis of the scattergrams and feature parameters showed that although these four samples were not significant in parameters such as PCT and D-Neu-SFL-W in the DIFF channel, they exhibited clear scattergram anomalies in the WNB channel, which were reflected in the N-APL-Ratio-YZ feature values in the RFC-S model.

Bleeding caused by disseminated intravascular coagulation, hypercoagulation, fibrinolysis, and thrombocytopenia is the leading cause of high ED rate among APL patients [7]. Therefore, PLT and PCT are important features in both the RFC-S model and the RFC-C model. This finding is similar to that of Rana et al. [14], who found that the inhibited platelet production and increased platelet destruction in APL patients lead to a decrease in PLT and PCT. The APL models in other studies always include the monocyte ratio, neutrophil count, lymphocyte count, or the CPD parameter NE-SFL to reflect the increase in immature granulocytes in the samples [13, 14, 18]. In contrast, the present study innovatively revealed that, owing to the different haemolytic reagents in the WNB channel, parameters such as *N-APL-Ratio-YZ* and *N-ClusterDist-YZ* in the WNB channel could effectively identify suspicious immature particle clusters with high fluorescence and relatively large volume in the WNB channel. By using the identified *D-APL-Ratio-XY* and *D-Neu-Area-XY*, cells with abundant cytoplasmic content within these suspicious immature particle clusters identified in the DIFF channel and considered “abnormal promyelocytes.” By continuously positioning suspicious particle clusters in different channels, we finally characterized the abnormal APL cells using the RFC-S model; this is a key reason why the RFC-S model is superior to other blood cell parameter models. Although the features extracted from 2D scatterplots have achieved excellent model performance in the current study, future other research could explore more robust high-dimensional metrics for feature computation, such as density-based metrics or graph-based topologies, to potentially capture additional morphological patterns.

Early detection is crucial to reduce the high ED rate among APL patients. Previous studies have shown that the timely diagnosis and management of APL in low-income countries using standardized guidelines and cooperative haematology laboratory networks can reduce ED by 50% and increase 3-year overall survival rate by 30% [23]. The RFC-S model in this study can achieve low-cost and efficient screening of APL because it uses simple and readily available parameters to generate screening results. While cross-platform validation of our model across haematology analyzers requires future study, the two-step methodology—combining deep learning feature extraction with interpretable machine learning—provides a framework for point-of-care screening. Implementation via integration into analyzer software could guide urgent treatment decisions in resource-limited settings, potentially reducing APL early mortality.

## Supplementary Information

Supplementary Material 1.

Supplementary Material 2.

## Data Availability

Data cannot be shared openly but are available on request from authors.

## Abbreviations

APL: Acute promyelocytic leukemia
RFC-S: Random forest classifier-scatterplot
RFC-C: Random forest classifier-cell population data
AML: Acute myeloid leukemia
ED: Early death
ATRA: All-trans retinoic acid
AI: Artificial intelligence
ANN: Artificial neural network
CNN: Convolutional neural network
CBC: Complete blood count
CPD: Cell population data
ALL: Acute lymphocytic leukaemia
HCs: Healthy controls
WHO: World Health Organization
SS: Side scatter
FL: Fluorescence
FS: Forward scatter
Grad-CAM: Radient-weighted class activation mapping
ROIs: Regions of interest
LR: Logistic regression
SVM: Support vector machine
LDA: Linear discriminant analysis
RFC: Random forest classifier
AdaBoost: Adaptive boosting
RFE: Recursive feature elimination
ROC: Receiver operating characteristic curve
AUC: Area under the Receiver Operating Characteristic Curve
SHAP: Shapley Additive Explanation

## References

[1] Döhner H, Weisdorf DJ, Bloomfield CD. Acute myeloid leukemia. N Engl J Med. 2015;373:1136–52. 26376137 10.1056/NEJMra1406184

[2] Li W. The 5(th) Edition of the World Health Organization Classification of Hematolymphoid Tumors. In: Li W. Leukemia. Brisbane (AU): Exon Publications Copyright: The Authors.; The authors confirm that the materials included in this chapter do not violate copyright laws. Where relevant, appropriate permissions have been obtained from the original copyright holder(s) and all original sources have been appropriately acknowledged or referenced. 2022.

[3] Jácomo RH, Melo RA, Souto FR, de Mattos ER, de Oliveira CT, Fagundes EM, et al. Clinical features and outcomes of 134 Brazilians with acute promyelocytic leukemia who received ATRA and anthracyclines. Haematologica. 2007;92:1431–2. 18024380 10.3324/haematol.10874

[4] Liu J, Zhu HH, Jiang H, Jiang Q, Huang XJ. Varying responses of PML-RARA with different genetic mutations to arsenic trioxide. Blood. 2016;127:243–50. 26537301 10.1182/blood-2015-04-637678

[5] Lo-Coco F, Avvisati G, Vignetti M, Thiede C, Orlando SM, Iacobelli S, et al. Retinoic acid and arsenic trioxide for acute promyelocytic leukemia. N Engl J Med. 2013;369:111–21. 23841729 10.1056/NEJMoa1300874

[6] Franck C, Byrjalsen I. Fractionation of elastase-type enzyme activity in biological fluids using a centrifugal analyser. Biol Chem Hoppe-Seyler. 1990;371:465–9. 2202332 10.1515/bchm3.1990.371.1.465

[7] Lehmann S, Ravn A, Carlsson L, Antunovic P, Deneberg S, Möllgård L, et al. Continuing high early death rate in acute promyelocytic leukemia: a population-based report from the Swedish adult acute leukemia registry. Leukemia. 2011;25:1128–34. 21502956 10.1038/leu.2011.78

[8] Österroos A, Maia T, Eriksson A, Jädersten M, Lazarevic V, Wennström L, et al. A risk score based on real-world data to predict early death in acute promyelocytic leukemia. Haematologica. 2022;107:1528–37. 35081688 10.3324/haematol.2021.280093 PMC9244824

[9] Tajvidi Asr R, Rahimi M, Hossein Pourasad M, Zayer S, Momenzadeh M, Ghaderzadeh M. Hematology and hematopathology insights powered by machine learning: shaping the future of blood disorder management. Iran J Blood Cancer. 2024;16(4):9-19.

[10] Ghaderzadeh M, Shalchian A, Irajian G, Sadeghsalehi H, Sabet B. Artificial intelligence in drug discovery and development against antimicrobial resistance: A narrative review. Iran J Med Microbiol. 2024;18(3):135-47.

[11] Ghaderzadeh M, Aria M, Hosseini A, Asadi F, Bashash D, Abolghasemi H. A fast and efficient CNN model for B-ALL diagnosis and its subtypes classification using peripheral blood smear images. Int J Intell Syst. 2022;37:5113–33.

[12] Park JH, Qiao B, Panageas KS, Schymura MJ, Jurcic JG, Rosenblat TL, et al. Early death rate in acute promyelocytic leukemia remains high despite all-trans retinoic acid. Blood. 2011;118:1248–54. 21653939 10.1182/blood-2011-04-346437 PMC3790946

[13] Alcazer V, Le Meur G, Roccon M, Barriere S, Le Calvez B, Badaoui B, et al. Evaluation of a machine-learning model based on laboratory parameters for the prediction of acute leukaemia subtypes: a multicentre model development and validation study in France. Lancet Digit Health. 2024;6:e323–33. 38670741 10.1016/S2589-7500(24)00044-X

[14] Haider RZ, Ujjan IU, Shamsi TS. Cell population data-driven acute promyelocytic leukemia flagging through artificial neural network predictive modeling. Transl Oncol. 2020;13:11–6. 31733590 10.1016/j.tranon.2019.09.009 PMC6859536

[15] Haider RZ, Ujjan IU, Khan NA, Urrechaga E, Shamsi TS. Beyond the in-practice CBC: the research CBC parameters-driven machine learning predictive modeling for early differentiation among leukemias. Diagnostics (Basel). 2022. 10.3390/diagnostics12010138. 35054304 10.3390/diagnostics12010138 PMC8774626

[16] Liao H, Xu Y, Meng Q, Mao Z, Qiao Y, Liu Y, et al. A convolutional neural network-based, quantitative complete blood count scattergram-mapping framework promptly screens acute promyelocytic leukemia with high sensitivity. Cancer. 2023;129:2986–98. 37254628 10.1002/cncr.34890

[17] Arber DA, Orazi A, Hasserjian R, Thiele J, Borowitz MJ, Le Beau MM, et al. The 2016 revision to the World Health Organization classification of myeloid neoplasms and acute leukemia. Blood. 2016;127:2391–405. 27069254 10.1182/blood-2016-03-643544

[18] Cheli E, Chevalier S, Kosmider O, Eveillard M, Chapuis N, Plesa A, et al. Diagnosis of acute promyelocytic leukemia based on routine biological parameters using machine learning. Haematologica. 2022;107:1466–9. 35199507 10.3324/haematol.2021.280406 PMC9152968

[19] Zhu J, Lemaire P, Mathis S, Ronez E, Clauser S, Jondeau K, et al. Machine learning-based improvement of MDS-CBC score brings platelets into the limelight to optimize smear review in the hematology laboratory. BMC Cancer. 2022;22:972. 36088307 10.1186/s12885-022-10059-8 PMC9464379

[20] Hwang SM, Nam Y. Complete blood count and cell population data parameters from the Abbott Alinity HQ analyzer are useful in differentiating myelodysplastic syndromes from other forms of cytopenia. Int J Lab Hematol. 2022;44:468–76. 34877795 10.1111/ijlh.13777

[21] Vinholt PJ. The role of platelets in bleeding in patients with thrombocytopenia and hematological disease. Clin Chem Lab Med. 2019;57:1808–17. 31465290 10.1515/cclm-2019-0380

[22] Raess PW, van de Geijn GJ, Njo TL, Klop B, Sukhachev D, Wertheim G, et al. Automated screening for myelodysplastic syndromes through analysis of complete blood count and cell population data parameters. Am J Hematol. 2014;89:369–74. 24276948 10.1002/ajh.23643

[23] Rego EM, Kim HT, Ruiz-Argüelles GJ, Undurraga MS, Uriarte Mdel R, Jacomo RH, et al. Improving acute promyelocytic leukemia (APL) outcome in developing countries through networking, results of the international consortium on APL. Blood. 2013;121:1935–43. 23319575 10.1182/blood-2012-08-449918

