# Supplementary material for: Development of a screening model for APL using cell population data and deep learning-extracted WBC scattergram features
Source: BMC Cancer. 2025 Nov 7;25:1725. doi: 10.1186/s12885-025-15034-7 (PMC12593920; doi:10.1186/s12885-025-15034-7)

**1.Proportion of particles in the feature region**

In Grad-CAM, a redder region indicates a higher contribution of the cell population in that region to the positive determination of APL. Therefore, the number of particles in a region directly reflects the density of abnormal cells in that region. The proportion of particles in the feature region can be calculated as a feature parameter to provide a quantitative indicator of abnormal cell proliferation in the disease-related region. Four particle proportions were calculated for the four ROI feature regions in the four scattergrams, as shown in Supplementary Table 1. Let the number of cells be
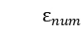
 and the total number of cells be
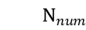
. The particle proportion for the feature region is calculated as follows:


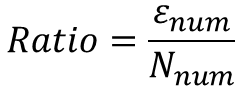


**2.Morphological characteristics of the feature region**

Due to the presence of abnormally differentiated blood cells (abnormal promyelocytes) in the peripheral blood of APL patients, the scattergram reveals abnormally large areas of different types of cell populations. Different cell populations intersect with APL-related ROIs, and changes in these particle populations related to ROIs may be related to the positive features of APL samples. Lymphocytes, monocytes, and neutrophils in the DIFF channel are related to APL ROIs, and white blood cells in the WNB channel are divided into two groups, both associated with APL ROIs.

Once the particle populations associated with the four ROIs are determined, the areas of these particle populations were calculated as feature parameters. The areas of the six particle populations were obtained from the two DIFF channel scattergrams, and the areas of the four particle populations were obtained from the two WNB channel scattergrams. The specific calculation steps are as follows: The target particle cluster for which the area needs to be calculated is denoised by means of Gaussian filter and threshold segmentation, the processed image is binarized, and the number of pixels in the calculated area is the area of the target area.


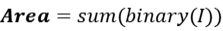


**3.Particle cluster spacing features**

Multiple particle clusters are usually distributed in the high-weight regions of the Grad-CAM map. The spacing between these clusters reflects the relative positional relationships between cells and the degree of distribution differences in volume, complexity, and nucleic acid content. The distance between the particle clusters was calculated to describe the differences in the distribution relationships between different cell populations, thereby describing the differences in the relative relationships between cell populations in the scattergrams of APL and non-APL patients. The three cell clusters in the two scattergrams of the DIFF channel yielded four particle cluster spacings, and two particle cluster spacings were calculated from the two WNB channel scattergrams, as shown in Supplementary Table 1. The distance was described by using the Euclidean distance between the centroids of the cell populations on the 2D scattergrams:


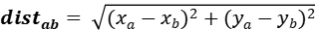

Supplement: Supplementary file 1 — Supplementary Material 1. [file 12885_2025_15034_MOESM1_ESM.docx]
