# Supplementary material for: Development of a screening model for APL using cell population data and deep learning-extracted WBC scattergram features
Source: BMC Cancer. 2025 Nov 7;25:1725. doi: 10.1186/s12885-025-15034-7 (PMC12593920; doi:10.1186/s12885-025-15034-7)
Supplement: Supplementary file 2 — Supplementary Material 2. [file 12885_2025_15034_MOESM2_ESM.docx]

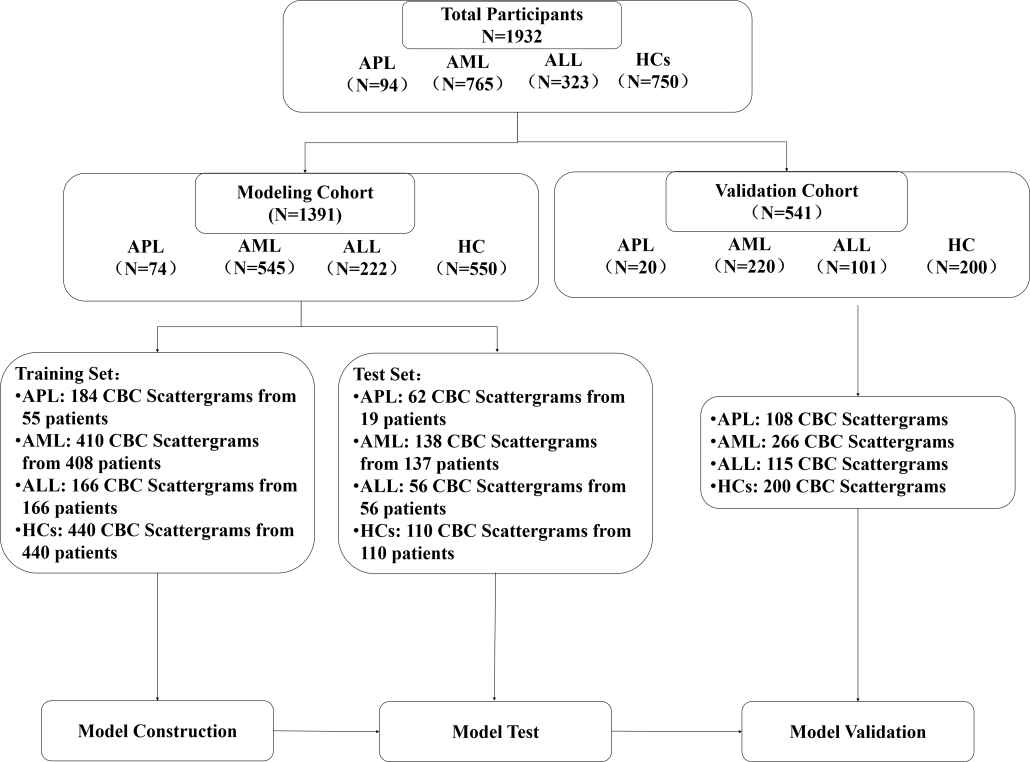


Supplementary Figure 1. The structure of the entire data set. AML, acute myeloid leukemia; APL, acute promyelocytic leukemia; ALL, acute lymphoblastic leukemia; HC, healthy controls; CBC, complete blood count.


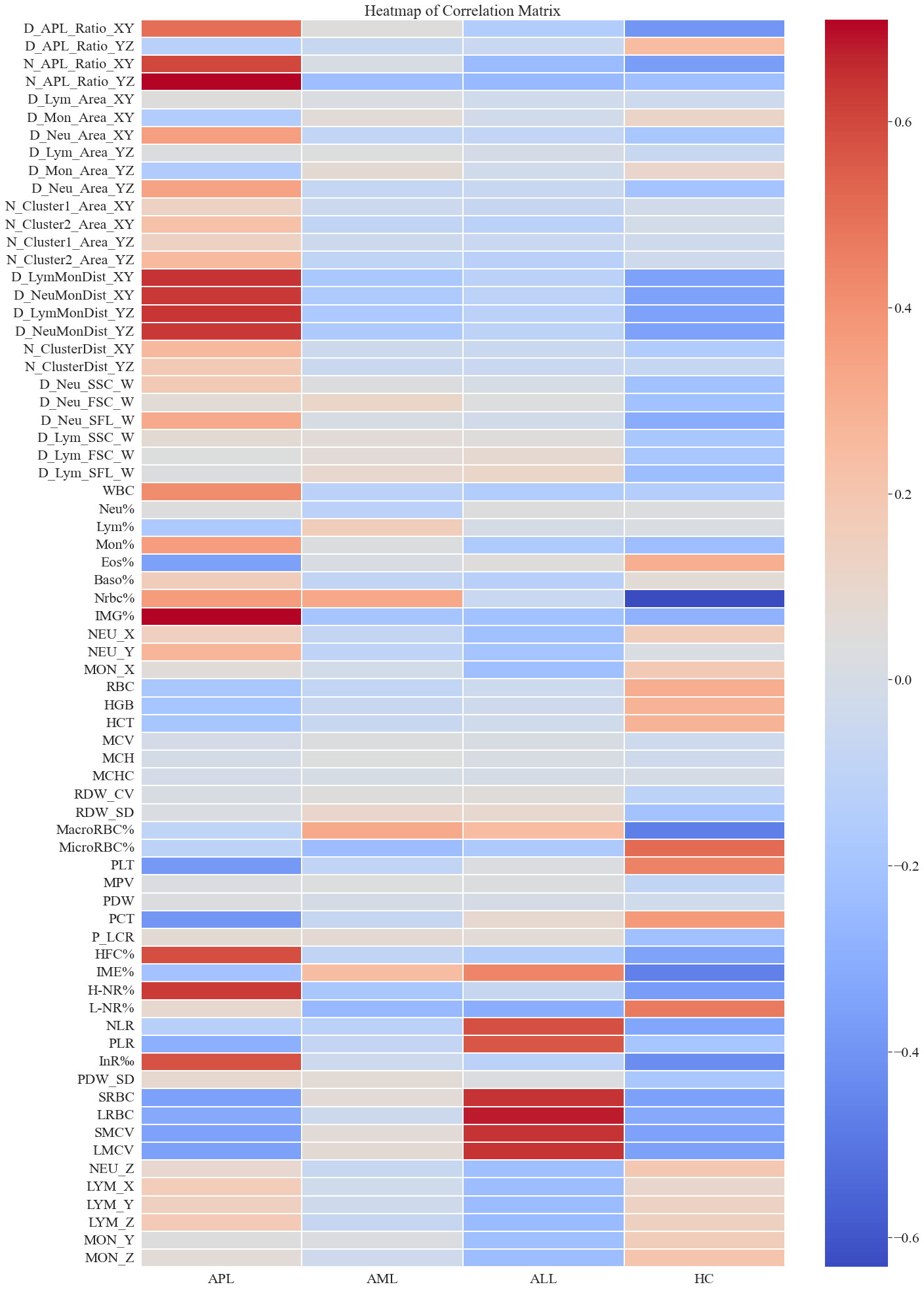


Supplementary Figure 2. Differences in scatterplot parameters in different groups of patients.

The heatmap of correlation matrix: color grading and clustering trends of 70 scattergram parameters among study groups. For heat map color grading ‘diverging Red to Blue’ scheme (for higher to lower values, respectively) was used. AML, acute myeloid leukemia; APL, acute promyelocytic leukemia; ALL, acute lymphoblastic leukemia; HC, healthy controls.


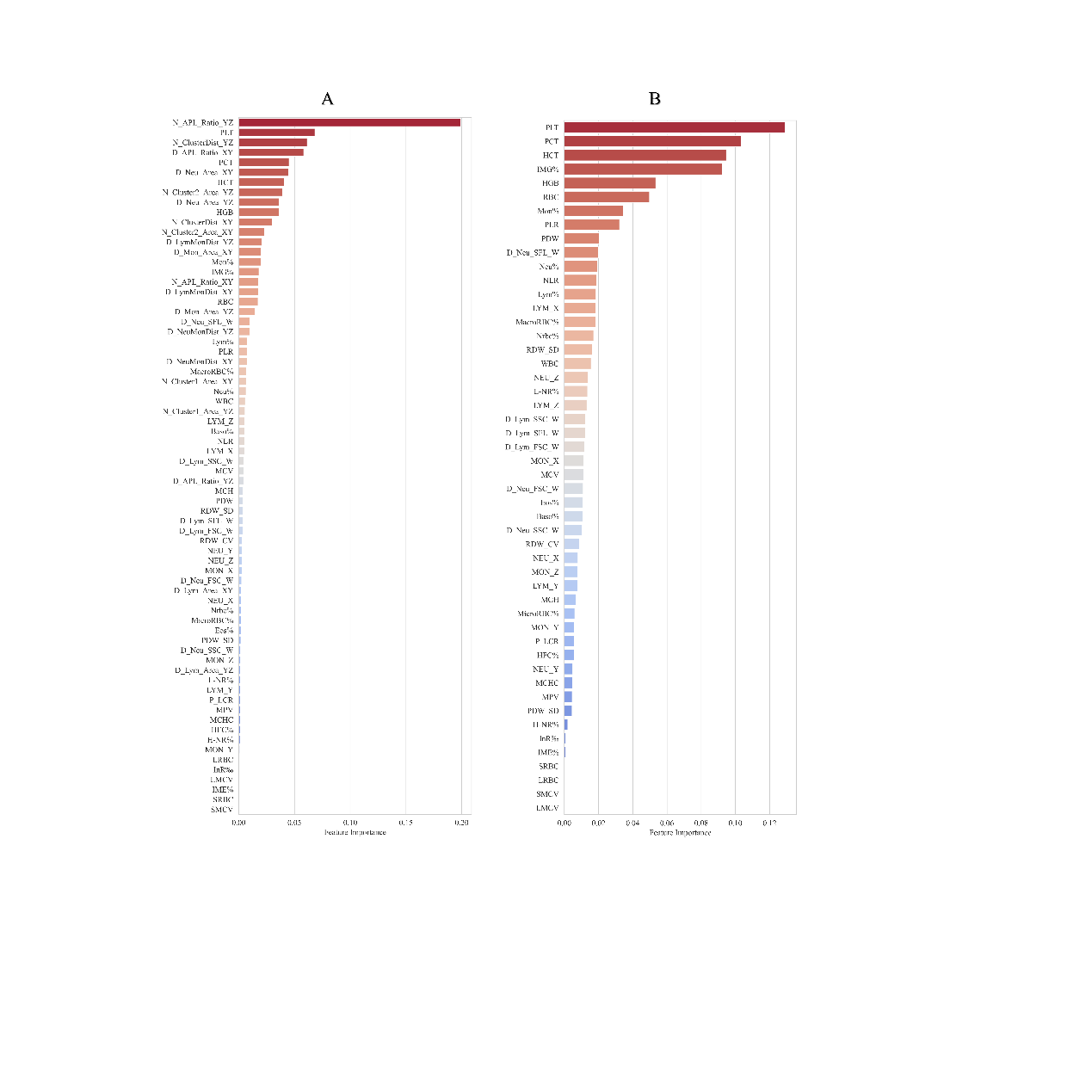


Supplementary Figure 3. Calculate feature importance ranking using the feature-importance function of the random forest model in the Python package, RFC-S (A) RFC-C (A).

Supplementary Table 1. 70 parameters of blood cell analysis

| Number | Abbreviations | Parameters |
| --- | --- | --- |
| 1 | WBC | White Blood Cell count |
| 2 | Neu% | Neutrophils percentage |
| 3 | Lym% | Lymphocytes percentage |
| 4 | Mon% | Monocytes percentage |
| 5 | Eos% | Eosinophils percentage |
| 6 | Baso% | Basophils percentage |
| 7 | Nrbc% | Nucleated red blood cell percentage |
| 8 | IMG% | Immature Granulocyte percentage |
| 9 | RBC | Red Blood Cell count |
| 10 | HGB | Hemoglobin Concentration |
| 11 | HCT | Hematocrit |
| 12 | PLT | Platelet count |
| 13 | MCV | Mean Corpuscular Volume |
| 14 | MCH | Mean Corpuscular Hemoglobin |
| 15 | MCHC | Mean Corpuscular Hemoglobin Concentration |
| 16 | RDW_CV | Red Blood Cell Distribution Width Coefficient of Variation |
| 17 | RDW_SD | Red Blood Cell Distribution Width Standard Deviation |
| 18 | MacroRBC% | Macrocyte percentage |
| 19 | MicroRBC% | Microcyte percentage |
| 20 | MPV | Mean Platelet Volume |
| 21 | PDW | Platelet Distribution Width |
| 22 | *PCT* | Plateletcrit |
| 23 | P_LCR | Platelet-large cell ratio |
| 24 | HFC% | High fluorescent Cell percentage |
| 25 | IME% | Immature eosinophil percentage |
| 26 | H-NR% | High forward scatter NRBC ratio, |
| 27 | L-NR% | Low forward scatter NRBC ratio |
| 28 | NLR | Neutrophil-to-lymphocyte ratio |
| 29 | PLR | Platelet-to-lymphocyte ratio |
| 30 | InR‰ | Infected Red Blood Cell permillage |
| 31 | PDW_SD | Platelet Distribution Width Standard Deviation |
| 32 | SRBC | Dimorphic population, smaller distribution RBC count |
| 33 | LRBC | Dimorphic population, larger distribution RBC count |
| 34 | SMCV | Dimorphic population, smaller distribution mean corpuscular volume |
| 35 | LMCV | Dimorphic population, larger distribution mean corpuscular volume |
| 36 | NEU_X | DIFF scattergram, mean neutrophil distribution-side scatter intensity |
| 37 | NEU_Y | DIFF scattergram, mean neutrophil distribution-side fluorescent light intensity |
| 38 | NEU_Z | DIFF scattergram, mean neutrophil distribution-forward scatter intensity |
| 39 | LYM_X | DIFF scattergram, mean lymophocyte distribution-side scatter intensity |
| 40 | LYM_Y | DIFF scattergram, mean lymophocyte distribution-side fluorescent intensity |
| 41 | LYM_Z | DIFF scattergram, mean lymophocyte distribution-forward scatter intensity |
| 42 | MON_X | DIFF scattergram, mean monocyte distribution-side scatter intensity |
| 43 | MON_Y | DIFF scattergram, mean monocyte distribution-side fluorescent light intensity |
| 44 | MON_Z | DIFF scattergram, mean monocyte distribution-forward scatter intensity |
| 45 | D_Neu_SSC_W | DIFF scattergram, mean neutrophil distribution-side scatter width |
| 46 | D_Neu_SFL_W | DIFF scattergram, mean neutrophil distribution-side fluorescent light width |
| 47 | D_Neu_FSC_W | DIFF scattergram, mean neutrophil distribution-forward scatter width |
| 48 | D_Lym_SSC_W | DIFF scattergram, mean lymophocyte distribution-side scatter width |
| 49 | D_Lym_SFL_W | DIFF scattergram, mean lymophocyte distribution-side fluorescent width |
| 50 | D_Lym_FSC_W | DIFF scattergram, mean lymophocyte distribution-forward scatter width |
| 51 | D_APL_Ratio_XY | DIFF scattergram SS-FL angle feature cell ratio |
| 52 | D_APL_Ratio_YZ | DIFF scattergram FS-FL angle feature cell ratio |
| 53 | N_APL_Ratio_XY | WNB scattergram SS-FL angle feature cell ratio |
| 54 | N_APL_Ratio_YZ | WNB scattergram FS-FL angle feature cell ratio |
| 55 | D_Lym_Area_XY | DIFF scattergram SS-FL angle lymophocyte distribution scatter area |
| 56 | D_Mon_Area_XY | DIFF scattergram SS-FL angle monocyte distribution scatter area |
| 57 | D_Neu_Area_XY | DIFF scattergram SS-FL angle neutrophil distribution scatter area |
| 58 | D_Lym_Area_YZ | DIFF scattergram FS-FL angle lymophocyte distribution scatter area |
| 59 | D_Mon_Area_YZ | DIFF scattergram FS-FL angle monocyte distribution scatter area |
| 60 | D_Neu_Area_YZ | DIFF scattergram FS-FL angle neutrophil distribution scatter area |
| 61 | N_Cluster1_Area_XY | WNB scattergram SS-FL angle cell cluster 1 area |
| 62 | N_Cluster2_Area_XY | WNB scattergram SS-FL angle cell cluster 2 area |
| 63 | N_Cluster1_Area_YZ | WNB scattergram FS-FL angle cell cluster 1 area |
| 64 | N_Cluster2_Area_YZ | WNB scattergram FS-FL angle cell cluster 2 area |
| 65 | D_LymMonDist_XY | DIFF scattergram SS-FL angle lymophocyte and monocyte distances |
| 66 | D_NeuMonDist_XY | DIFF scattergram SS-FL angle neutrophil and monocyte distances |
| 67 | D_LymMonDist_YZ | DIFF scattergram FS-FL angle lymophocyte and monocyte distances |
| 68 | D_NeuMonDist_YZ | DIFF scattergram FS-FL angle neutrophil and monocyte distances |
| 69 | N_ClusterDist_XY | WNB scattergram SS-FL angle cell cluster distance |
| 70 | N_ClusterDist_YZ | WNB scattergram FS-FL angle cell cluster distance |
